# Notices

**Published:** 1861-03

**Authors:** 


**NOTICES.**—*The Presbyterian Historical Almanac and Annual Remembrancer of the Church for* 1861, volume 3, 8vo, 328 pages, by Joseph M. Wilson, 111 South-Tenth street, Philadelphia, is sent post-paid for $1.12. It contains an alphabetical list of all the ministers of the whole Presbyterian family, with post-office address, and a vast amount of most valuable historical and statistical matter, exceedingly convenient and valuable, not only for clergymen, but for educated Presbyterian families. As many clergymen can not spare the dollar conveniently, we will send it post-paid to any minister who will forward us three new subscriptions to HALL'S JOURNAL OF HEALTH, of one dollar each.

*The Chemist and Druggist*, a monthly trade circular, 24 Bow Lane, Cannon street, West, London. Five shillings per annum, post free. Every druggist in the Union would find it to his interest to take this publication.

